# Crystallization Control of *N,N′*-Dioctyl Perylene Diimide by Amphiphilic Block Copolymers Containing poly(3-Hexylthiophene) and Polyethylene Glycol

**DOI:** 10.3389/fchem.2021.699387

**Published:** 2021-06-10

**Authors:** Xiaohui Yang, Wanlong Lu, Jingning Cao, Chenyang Zhai, Weili Li, Fangwen Zha, Guanghao Lu, Hongkun Tian, Demei Yu, Laju Bu

**Affiliations:** ^1^ School of Chemistry, MOE Key Laboratory for Nonequilibrium Synthesis and Modulation of Condensed Matter, and Xi’an Key Laboratory of Sustainable Energy Material Chemistry, Xi’an Jiaotong University, Xi’an, China; ^2^ Frontier Institute of Science and Technology, and State Key Laboratory of Electrical Insulation and Power Equipment, Xi’an Jiaotong University, Xi’an, China; ^3^ State Key Laboratory of Polymer Physics and Chemistry, Changchun Institute of Applied Chemistry, Chinese Academy of Sciences, Changchun, China

**Keywords:** crystallization modifiers, amphiphilic block copolymers, sonocrystallization, hydrosol, *N,N*′-dioctyl perylene diimide

## Abstract

The preparation of micron- to nanometer-sized functional materials with well-defined shapes and packing is a key process to their applications. There are many ways to control the crystal growth of organic semiconductors. Adding polymer additives has been proven a robust strategy to optimize semiconductor crystal structure and the corresponding optoelectronic properties. We have found that poly(3-hexylthiophene) (P3HT) can effectively regulate the crystallization behavior of *N,N′*-dioctyl perylene diimide (C8PDI). In this study, we combined P3HT and polyethylene glycol (PEG) to amphiphilic block copolymers and studied the crystallization modification effect of these block copolymers. It is found that the crystallization modification effect of the block copolymers is retained and gradually enhanced with P3HT content. The length of C8PDI crystals were well controlled from 2 to 0.4 μm, and the width from 210 to 35 nm. On the other hand, due to the water solubility of PEG block, crystalline PEG-*b*-P3HT/C8PDI micelles in water were successfully prepared, and this water phase colloid could be stable for more than 2 weeks, which provides a new way to prepare pollution-free aqueous organic semiconductor inks for printing electronic devices.

**GRAPHICAL ABSTRACTS:**
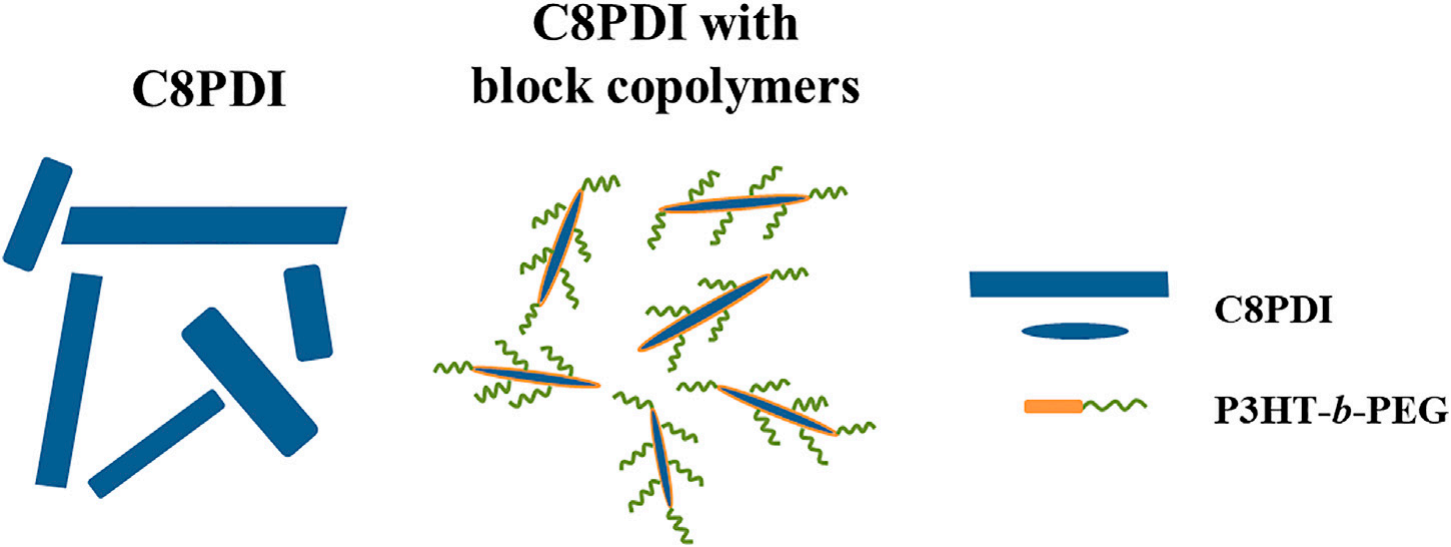


## Introduction

The preparation of micron- and nanometer-sized functional materials with well-defined shapes and sizes has broad applications in catalysis, electronics, medicine and other fields. Controlling the crystal morphology of organic semiconductors has always been a research focus due to their morphology impact on the optoelectronic properties. The existing methods for controlling the growth of crystals include melting techniques, vapor techniques, solution-processing, chemical reaction and patterning (He et al., 2014; Wang et al., 2018). Solution-processing methods include recrystallization, dip coating, drop casting, solvent exchange, solvent vapor diffusion, *etc* (Diao et al., 2014). In the solution-processing method, the growth of the crystals is closely related to factors such as solvent, solubility, concentration, temperature, pressure, *etc* (Wang et al., 2018). Therefore, through adding solvent additives to the semiconductor solution, thermal annealing or solvent annealing of the semiconductor film, polar solvent treatment of the semiconductor film, and chemical modification of the semiconductor molecular structures, the morphology of the semiconductor crystals can be effectively improved (Huang et al., 2014).

In addition, polymer additives can effectively control the orientation and morphology of organic semiconductor crystals (He et al., 2019). The morphology of crystal is usually controlled by changing the ratio of polymer to semiconductor, concentration, solvent and other conditions. P3HT is a widely studied p-type polymer. Bu *et al.* found that P3HT can control the crystallization behavior of the n-type semiconductor small molecule *N*,*N*′-dioctyl perylene diimide (C8PDI), making fibrillar C8PDI crystals (Bu et al., 2012). P3HT can also control the crystal morphology of other small semiconductor molecules such as diphenyl-naphthalenediimide (DPNDI) (Xu et al., 2018) and 2,5-di-(2-ethylhexyl)-3,6-bis (5″-*n*-hexyl-2,2′,5′,2′′] terthiophen-5-yl)-pyrrole [3,4-*c*] pyrrole-1,4-dione (SMDPPEH) (Bi et al., 2019). 6,13-bis(triisopropylsilylethynyl) pentacene (TP) is a solution processable p-type organic semiconductor with high hole mobility. The addition of P3HT can transform TP from disorderly arranged large needle-shaped crystals into curved thin lines, enhancing its long-range ordering (Chen et al., 2013). The addition of polyacrylate, polyethylene glycol (PEG) and other polymers can also improve the molecular orientation of TP and enhance its long-range ordering (He et al., 2013; He et al., 2020). Huang *et al.* used the amphiphilic block copolymer PEG-block-poly(ethyleneimine) (PEG-*b*-PEI) as the additive of a perylene derivative (Huang et al., 2016). By adjusting the concentration of the block copolymer and the pH of the solution, a series of perylene derivative with different crystal morphologies can be obtained. In addition, polymer additives can also improve the uniformity of the semiconductor film morphology, and induce phase separation between the semiconductor molecule and the polymer (Asare-Yeboah et al., 2016; Da Rocha et al., 2018). Therefore, adding polymer additives is a widely used and robust strategy to regulate the crystal structure of semiconductors.

Block copolymers can combine the superior properties of different polymers, and also produce various phase structures and self-assembly morphologies due to microphase separation (Kim et al., 2010). PEG has flexible mechanics and good solubility in water, alcohol and other green solvents. Here, we use block copolymer systems (combining P3HT with PEG) to regulate the C8PDI crystallization, hoping that the block copolymers can bring some new properties while maintaining the crystal modification effect of P3HT on C8PDI. By keeping PEG at the same molecular weight, a series of block copolymers containing different numbers of P3HT fragments were synthesized. We first explored the regulation effect of different block copolymers on C8PDI crystallization and found that the crystallization modification effect is retained and gradually enhanced as P3HT content increases, making the length and width of C8PDI crystals gradually decrease. Then we prepared stable crystalline P3HT-*b*-PEG/C8PDI micelles in water by extracting their micelles in toluene (Tol) with water, which provides a new way to prepare pollution-free aqueous organic semiconductor inks for printing electronic devices.

## Results and Discussion

**SCHEME 1:**
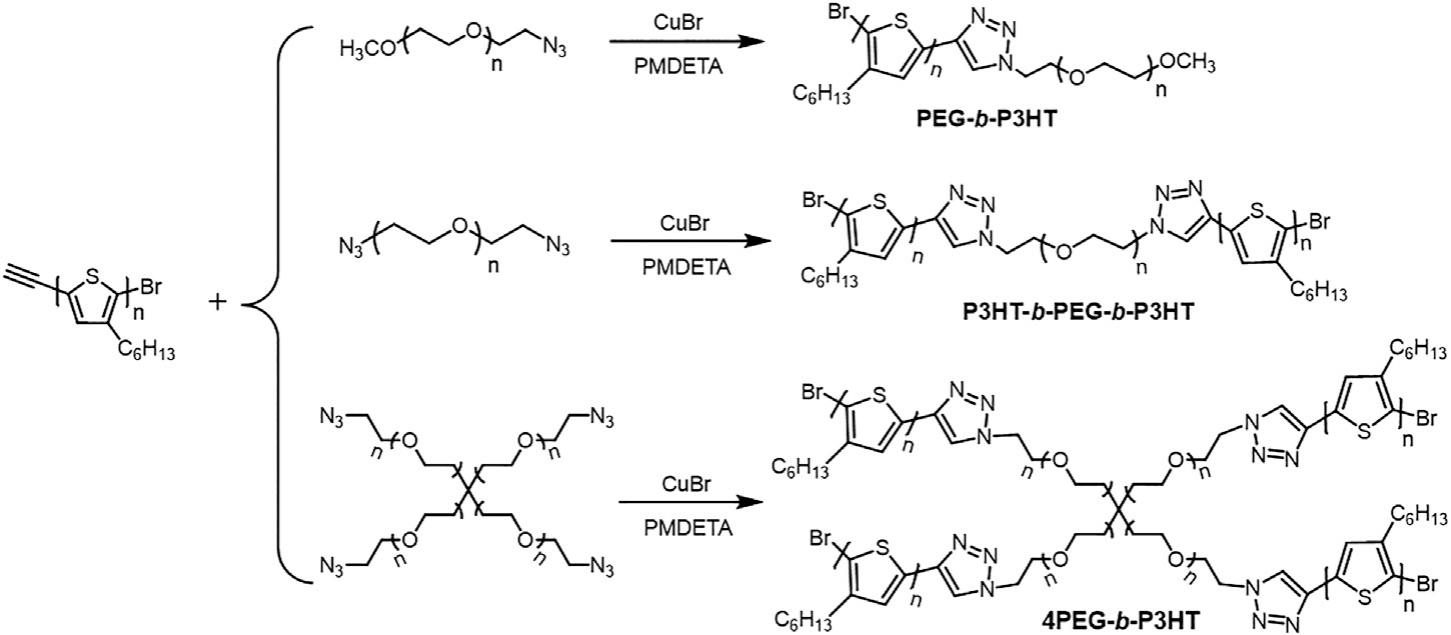
Synthetic routes of block copolymers.

**Synthesis of block copolymers.** Three block copolymers were synthesized by cycloaddition click reaction between monoethynyl-terminated P3HT (EP3HT) and azide-terminated PEG (azide-PEG) by copper-catalyzed azide-alkyne, shown in Scheme 1 (Kempf et al., 2013). The specific experimental steps are shown in the experimental section. EP3HT was synthesized according to the reported method (Jeffries-El et al., 2005; Miyakoshi et al., 2005). The product was purified by column chromatography and recycling preparative liquid chromatography. ^1^H nuclear magnetic resonance (NMR) spectroscopy shows the regioregularity of EP3HT is 90% (Supplementary Figure S1). According to the gel permeation chromatography (GPC), the number average molecular weight (M_n_) of EP3HT is 2,800 g/mol and the polymer dispersity index (PDI) is 1.15 (Supplementary Figures S2, S3, S4). The M_n_ and PDI of the EP3HT are also determined to be 1800 g/mol and 1.01, respectively, by matrix-assisted laser desorption ionization time-of-flight mass spectroscopy (MALDI-TOF-MS) (Supplementary Figure S5). The ratio of monoethynyl end-capped EP3HT is 95% and the double ethynyl end-capped EP3HT is 5%, which is calculated from the MALDI-TOF-MS spectrum. We purchased three kinds of azide-PEGs with different structures but same weight average molecular weight (M_w_). The azide-PEGs were purified by sedimentation in ether. Their structures were characterized by ^1^H NMR and fourier transform infrared spectroscopy (FT-IR) spectroscopy (Supplementary Figure S6) and their molecular weights were measured by GPC. These three kinds of azide-PEGs were coupled with the synthesized EP3HT to generate P3HT-*b*-PEG, P3HT-*b*-PEG-*b*-P3HT and four-armed P3HT-*b*-PEG (4P3HT-*b*-PEG) respectively by copper-catalyzed azide-alkyne cycloaddition click reaction. The success of block copolymers synthesis was confirmed by ^1^H NMR spectroscopy and the molecular weights measured by GPC.

**FIGURE 1:**
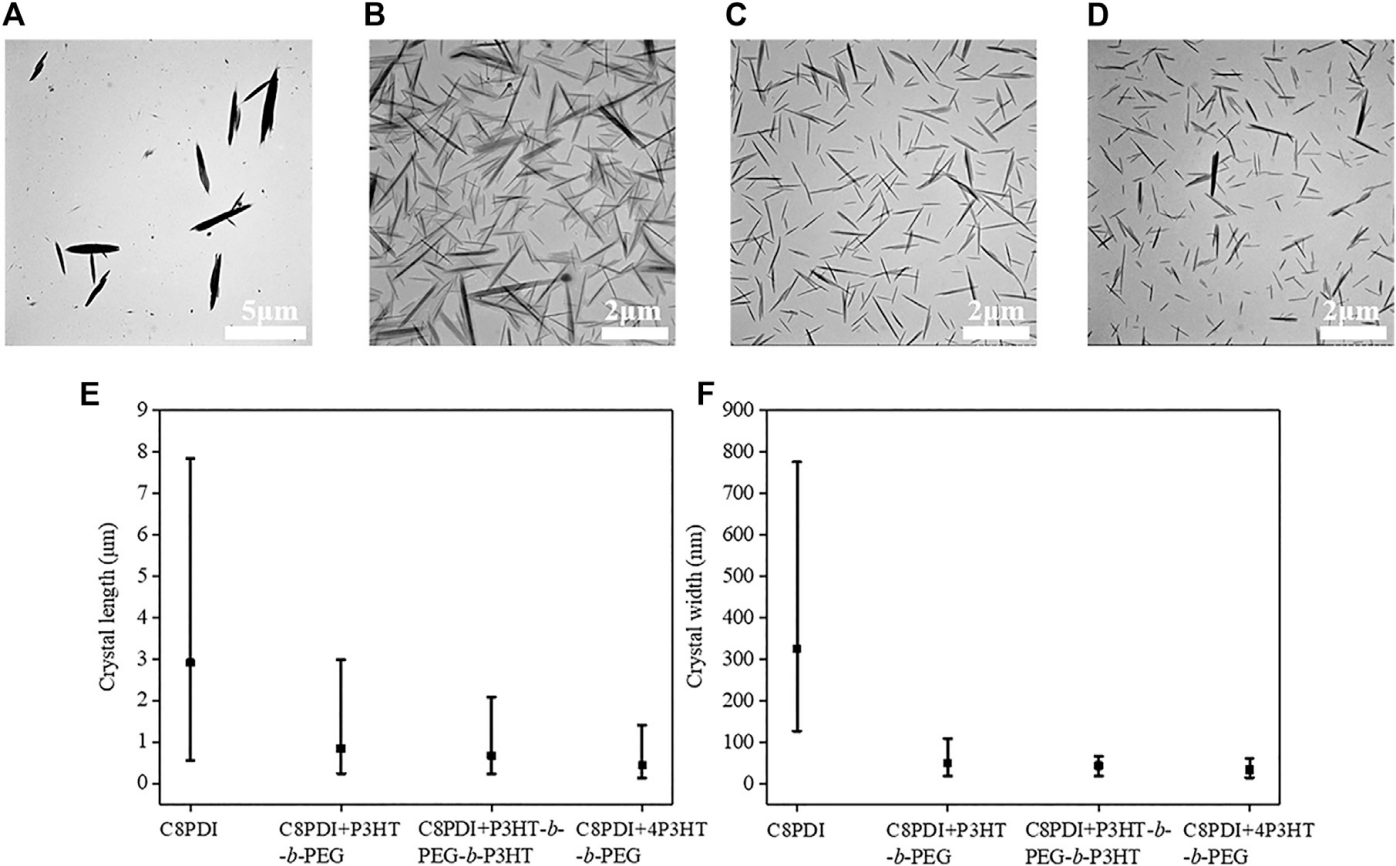
TEM images of C8PDI crystals sonocrystallized from 0.5 mg/ml *o*-DCB solution without **(A)** and with 1.0 mg/ml block copolymers, and the block copolymers are P3HT-*b*-PEG **(B)**, P3HT-*b*-PEG-*b*-P3HT **(C)** and 4P3HT-*b*-PEG **(D)**. The length **(E)** and width **(F)** statistics charts of the corresponding C8PDI crystals.

**Self-Assembly in *o*-dichlorobenzene (*o*-DCB).** To investigate the crystallization modification effect of block copolymers, we compared the crystallization behavior of C8PDI without and with block copolymers. 1.0 mg/ml C8PDI supersaturated solution in *o*-DCB was prepared by heating at 120°C for hours. Before precipitation, the solution was put into ultrasound for 2 h. Yellow clear solution changed to brick red suspension soon after putting in the sonication bath. The resulted crystals show an average length of 2.91 μm and an average width of 0.33 μm, as shown in Figure 1. The control experiment was done with 2.0 mg/ml P3HT-*b*-PEG and 1.0 mg/ml C8PDI in *o*-DCB. The supersaturated solution of C8PDI with P3HT-*b*-PEG is in orange color and clear for hours after cooled to room temperature, while the C8PDI supersaturated solution without block copolymers precipitates soon after cooling. During sonication, the C8PDI with P3HT-*b*-PEG solution changes to wine red color and remains clear. By radiating the solution with a red laser, distinct Tyndall scattering was observed which means colloid is formed (Supplementary Figure S7). After checking the colloid with TEM (shown in Figure 1B), needle-like micelles were observed. The micelles might be constructed of C8PDI crystals in the core and surrounded by P3HT-*b*-PEG layers.

By replacing the diblock copolymer P3HT-*b*-PEG with triblock copolymer and dendritic star copolymer, colloids with distinct Tyndall scattering were also obtained. From the TEM results shown in Figure 1, all the three colloids are composed of needle-like micelles. As the number of P3HT block increases, the average length of the needle-like micelles decreases from 0.83, to 0.67 and 0.44 μm, and the average width decreases from 50 to 44 and 34 nm. It is noted that the size of C8PDI crystals from the colloid of 4P3HT-*b*-PEG and C8PDI is an order of magnitude smaller than pure C8PDI crystals both in length and width.

It can be clearly seen that, blending C8PDI with block copolymers containing P3HT segments and using the ultrasound treatment can modify the C8PDI crystals morphology, making the C8PDI crystals shorter, thinner, more dispersed and more uniform, which suggests that the crystallization modification effect of the copolymers on C8PDI was gradually strengthened. The block copolymers would adsorb on ends and sides of the C8PDI crystal planes, thereby inhibiting the longitudinal and lateral growth of the C8PDI crystals. At the same time, ultrasound promotes crystallization and makes crystals more uniform (Kurotani et al., 2009). These results prove that block copolymers containing P3HT segments have regulation effect on C8PDI crystals and provides a new idea for adjusting the crystal morphology of organic semiconductors.

**FIGURE 2:**
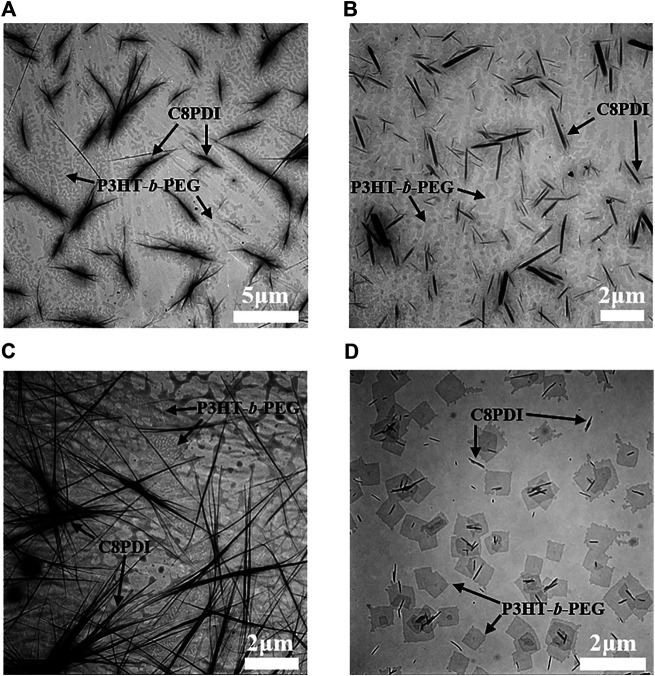
TEM images of P3HT-*b*-PEG and C8PDI drop-cast from *o*-DCB solution (10:1) before **(A)** and after **(B)** ultrasound treatment. TEM images of P3HT-*b*-PEG and C8PDI drop-cast from Tol solution (10:1) before **(C)** and after **(D)** ultrasound treatment.

**Crystallization in different solvents.** The crystallization of diblock copolymer and C8PDI in different solvents was also explored. Using *o*-DCB and Tol as solvents, respectively, solutions of diblock copolymer P3HT-*b*-PEG and C8PDI were prepared. The concentration ratio of P3HT-*b*-PEG to C8PDI is both 10:1. Similarly, heating was applied to completely dissolve the solute, and then the solutions were exposed to ultrasound for 2 h. The crystals morphology before and after the ultrasound treatment were shown in Figure 2. C8PDI crystals were fibrous before the ultrasound treatment both in *o*-DCB and Tol, and after the ultrasound treatment, C8PDI changed to rod-like crystals with much smaller size. As for the diblock copolymer P3HT-*b*-PEG, the crystals were seaweed-like in *o*-DCB both before and after the ultrasound treatment. While in Tol, they were dendritic-like crystals before the ultrasound treatment and changed to square flake crystals after the ultrasound treatment. The dendritic and seaweed crystals of the diblock copolymer were both caused by the PEG block crystallization (Zhai et al., 2006), and the square flake crystal is the typical single crystal morphology of PEG (Jiang et al., 2014). Supplemenatry Figure S8 shows the regular square crystals of pure P3HT-*b*-PEG and pure PEG-N_3_ in Tol after the ultrasound treatment. In summary, the diblock copolymer and C8PDI have different crystal morphologies in different solvents.

**FIGURE 3:**
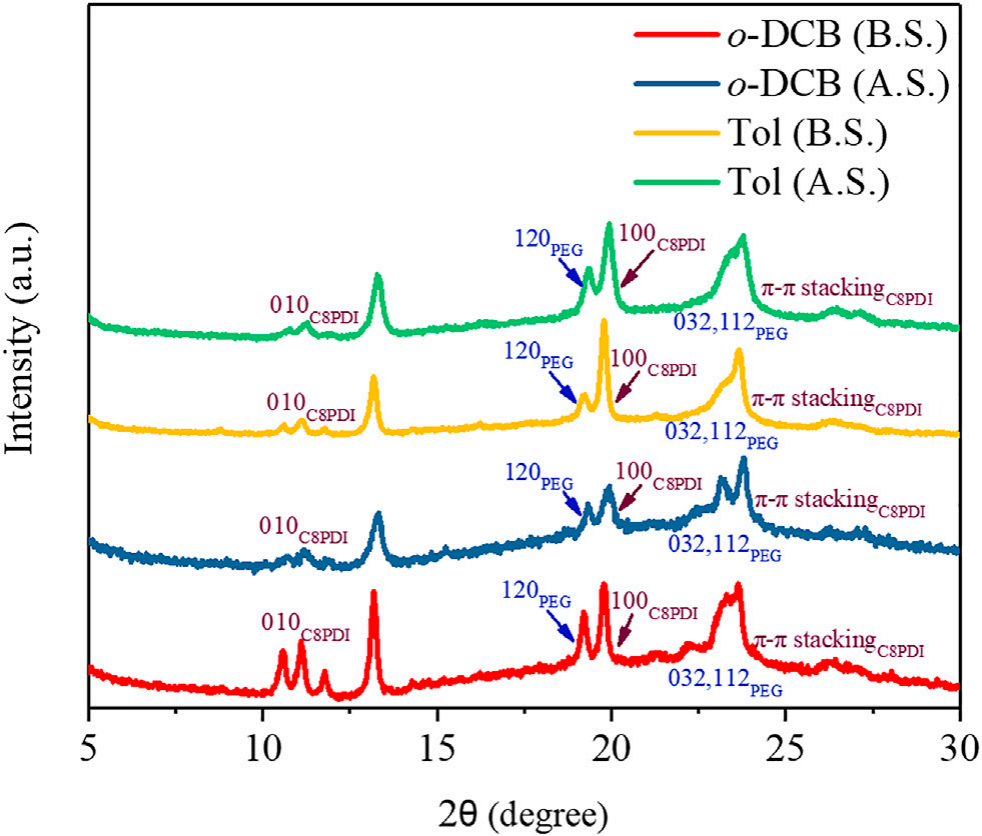
XRD patterns of P3HT-*b*-PEG and C8PDI (10:1) in *o*-DCB and Tol before sonocrystallization (B.S.) and after sonocrystallization (A.S.).

X-ray diffraction (XRD) experiments were performed on the films corresponding to the four samples in Figure 2 and the XRD patterns is shown in Figure 3. When P3HT-*b*-PEG and C8PDI were in *o*-DCB, they exhibited multiple distinct diffraction peaks at 2 θ values 11.1, 13.2, 19.2, 19.8, 23.2, and 23.7° before the sonocrystallization. The peaks at 11.1, 13.2, 19.8, and 23.7° result from the C8PDI crystals. The peaks at 19.8 and 11.1° correspond to *d*-spacing 4.48 and 7.97 Å, respectively, corresponding to the lattice parameters a (along the length) and b (along the width), respectively (Ameen et al., 2013). And the peak at 23.7° corresponds to *d*-spacing 3.75 Å, which is a typical intermolecular distance of perylene molecule along the π-π stacking direction (Ameen et al., 2013). Two primary characteristic diffraction peaks of crystalline PEG phase at 2θ = 19.2 and 23.2° were also observed (Jiang et al., 2008), corresponding to *d*-spacing 4.62 and 3.83 Å, respectively. The peaks at 19.2 and 23.2° correspond to the 120, and 032 and 112 crystal planes of monoclinic PEG crystals, respectively (Zhang et al., 2009). After the sonocrystallization, there is no significant change in diffraction peaks compared with before the sonocrystallization. When P3HT-*b*-PEG and C8PDI were in Tol, they showed diffraction peaks at the same positions as in *o*-DCB, indicating that changing the solvent from *o*-DCB to Tol or ultrasound treatment didn’t change the crystal structure of P3HT-*b*-PEG and C8PDI and the different crystal morphology of P3HT-*b*-PEG and C8PDI in *o*-DCB and Tol was caused by dynamics.

**FIGURE 4:**
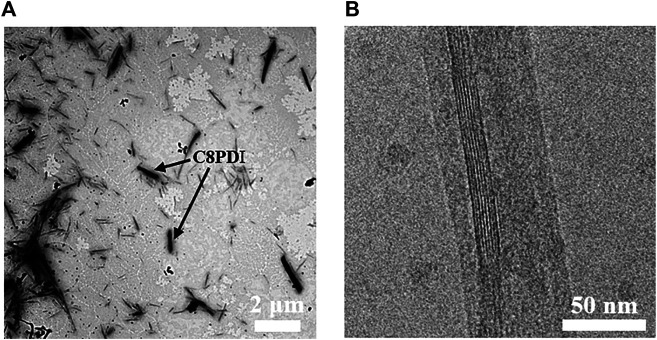
TEM image **(A)** and cryo-TEM image **(B)** of P3HT-*b*-PEG and C8PDI drop-cast from water colloid which was extracted from sonicated Tol solution (P3HT-*b*-PEG: C8PDI = 10:1).

**Preparation of micelles of diblock copolymer and C8PDI in water.** By extracting the Tol solution of diblock copolymer P3HT-*b*-PEG and C8PDI (10:1) with water, crystalline P3HT-*b*-P3HT/C8PDI micelles in water were successfully prepared. The specific method is first adding water to the Tol solution after ultrasonic crystallization and vigorously shaking the solution. After letting the solution stand for a period of time, the solution could be clearly divided into two layers. The upper layer, Tol phase, was emulsion, and the lower water phase was light yellow, clear, transparent (Supplementary Figure S7B). The water phase had the Tyndall effect, so it was a hydrosol. (Supplementary Figure S7C). By observing the crystal morphology in the hydrosol, it is proved that the diblock copolymer and C8PDI have been successfully extracted from the Tol phase into the water phase. From the TEM image (Figure 4A), the short and rod-like C8PDI crystals and dendritic P3HT-*b*-PEG crystals can be seen. Figure 4B is a cryo-TEM image, from which the lattice of C8PDI crystal can be clearly seen. What’s more, the hydrosol of P3HT-*b*-PEG and C8PDI can be stable for more than 2 weeks. The successful extraction is mainly attributed to the high solubility of PEG block in water. The solubility of the diblock copolymer in Tol is greater than that of C8PDI. Therefore, after ultrasound treatment, the diblock copolymer and C8PDI will form a micellar structure with P3HT-*b*-PEG as the coronal and C8PDI as the core. Since PEG is soluble in water, the micelles of diblock copolymer and C8PDI are soluble in water. Therefore, after adding water, the micelles will transfer to the water phase and finally reach a distribution equilibrium between Tol and water. We also used other copolymers to do this experiment but the results were not as good as P3HT-*b*-PEG. As for P3HT-*b*-PEG, it is noted that changing the concentration ratio of P3HT-*b*-PEG to C8PDI to 8:1 or 6:1, stable nanocrystalline micelles of diblock copolymer and C8PDI in water can be still successfully obtained by the method we described above.

**FIGURE 5:**
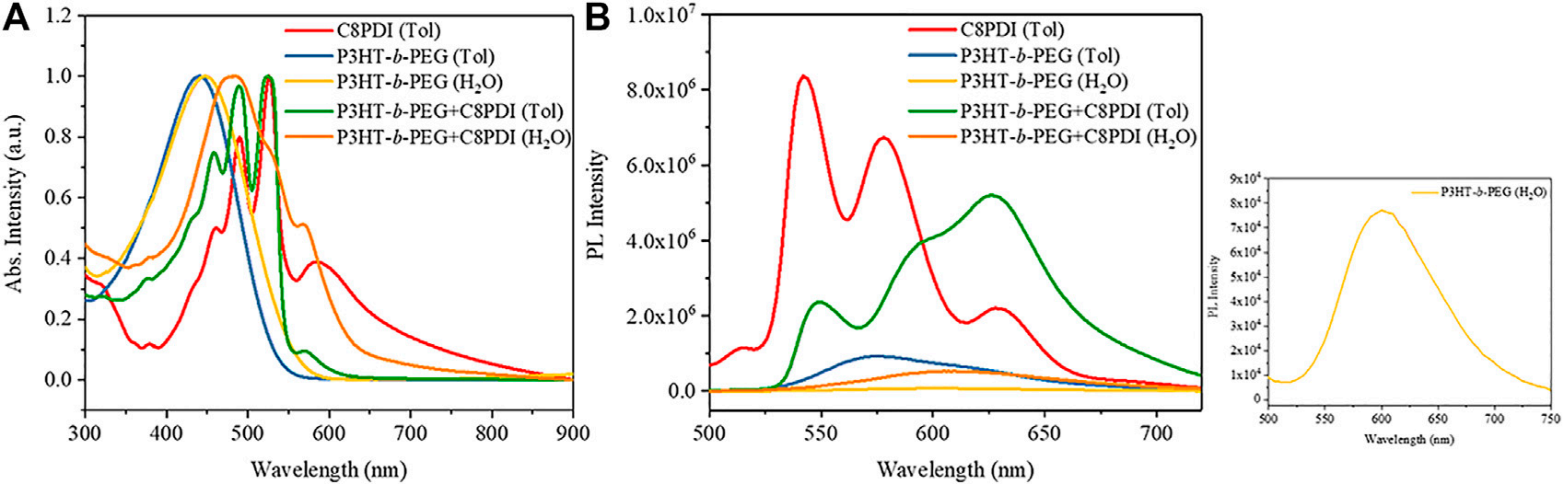
Normalized UV−vis spectra **(A)**and PL emission spectra **(B)** of C8PDI nanocrystals suspended in Tol noted as C8PDI (Tol), P3HT-*b*-PEG in Tol after ultrasound noted as P3HT-*b*-PEG (Tol), P3HT-*b*-PEG colloid in water extracted from its sonicated Tol solution noted as P3HT-*b*-PEG (H_2_O), P3HT-*b*-PEG and C8PDI (10:1) colloid in Tol noted as P3HT-*b*-PEG + C8PDI (Tol), and P3HT-*b*-PEG and C8PDI colloid in water extracted from their sonicated Tol solution noted as P3HT-*b*-PEG + C8PDI (H_2_O). PL emission spectra were collected using an excitation wavelength of 473 nm.

The UV-vis absorption spectra of solutions or colloidal solutions (Figure 5A) also prove this phenomenon. The UV−vis spectrum of P3HT-*b*-PEG colloid in Tol after ultrasound (the blue line) shows an absorption band ranging from 300 to 550 nm with a maximum at about 420 nm (Reichstein et al., 2016). The absorption of P3HT-*b*-PEG colloid in water (the yellow line, extracted from its sonicated Tol solution) is redshifted by 10 nm compared with that in Tol. C8PDI nanocrystals in sonicated Tol solution (the red line) shows absorption between 400–700 nm (Le et al., 2018). The peak at about 590 nm is due to the assembly of C8PDI molecules. The absorption of P3HT-*b*-PEG and C8PDI colloid in sonicated Tol solution (the green line) shows an addition absorption of C8PDI and P3HT-*b*-PEG. The absorption at 580 nm corresponds to the absorption at 590 nm of the C8PDI nanocrystals in sonicated Tol solution. P3HT-*b*-PEG and C8PDI colloid in water extracted from their Tol solution (the orange line) shows an absorption band ranging from 400 to 650 nm with the maximum absorption peak at 480 nm and two shoulder peaks at 530 and 580 nm, respectively. The shoulder peak at 580 nm resulting from the assembly of C8PDI proves the success of extracting C8PDI from the Tol solution into the water.

Photoluminescence (PL) measurement was also used to explore the interaction between P3HT-*b*-PEG and C8PDI. We prepared C8PDI (0.2 mg/ml), P3HT-*b*-PEG (2 mg/ml) and P3HT-*b*-PEG: C8PDI (2:0.2) Tol solutions because pure C8PDI has low solubility in Tol even at high temperature. Similarly, heat was applied to completely dissolve the solute and then the solutions were exposure to ultrasound. After the ultrasound, the PL spectra of the three samples were measured. And the P3HT-*b*-PEG: C8PDI (2:0.2) colloidal solution and P3HT-*b*-PEG (2 mg/ml) colloidal solution were extracted by water, then the PL of these two water phases were also measured. The results were shown in Figure 5B. The typical PL spectrum of C8PDI has three main peaks from 530 to 650 nm (Boobalan et al., 2014) and P3HT-*b*-PEG has a maximum PL intensity at ∼576 nm which is characteristic of regioregular P3HT in good solvent (McCullough, 1998). The PL intensity of C8PDI was much higher than that of P3HT-*b*-PEG, and the PL of the blend solution had a middle intensity between P3HT-*b*-PEG and C8PDI. In other words, P3HT-*b*-PEG partially quenched the PL of C8PDI, indicating that there is energy transfer between P3HT-*b*-PEG and C8PDI. After the extraction, pure P3HT-*b*-PEG in water, and P3HT-*b*-PEG and C8PDI in water had very weak fluorescence, and the maximum PL intensity is at 600 and 620 nm, respectively.

## Materials and Methods

### Materials

2,5-dibromo-3-hexylthiophene, isopropylmagnesium chloride (2.0 M solution in THF), ethynylmagnesium bromide (0.5 M solution in THF) and deuterated chloroform were purchased from Energy Chemical. Copper (I) bromide was purchased from aladdin. *N*,*N*,*N*′,*N*′,*N*″-pentamethyldiethylenetriamine (PMDETA) was purchased from Innochem. 1,3-bis(diphenylphosphino)-propanenickel (II) chloride [Ni(dppp)Cl_2_] was purchased from Acros. Neutral alumina was purchased from Shanghai Weiting. Deionized water was purchased from Xi’an Dongguan. Tetrahydrofuran (THF), dichloromethane, methanol, diethyl ether, hexane, chloroform, Tol, *o*-DCB were purchased from Chengdu Kelong. THF was freshly distilled prior to use from sodium/benzophenone under argon. Dichloromethane was freshly distilled prior to use from calcium hydride under argon. Diethyl ether was dried with anhydrous sodium sulfate. Azide-PEGs mPEG-N_3_ (M_w_ = 20,000), N_3_-PEG-N_3_ (M_w_ = 20,000) and 4Arm-PEG-N_3_ (M_w_ = 20,000) were purchased from Meiluo Technology. C8PDI was purchased from Ogantec.

### Instrumentation

^1^H-NMR spectra were obtained on an Ascend^™^ 400 spectrometer at room temperature. The solvent in measurement was deuterated chloroform. GPC measurements were carried out at room temperature on a PL-GPC 220 using THF as eluent at a flow rate of 1ml/min at room temperature. MALDI-TOF mass measurements were performed on a Bruker autoflex III smartbeam MALDI-TOF/TOF mass spectrometer using 2-[(2E)-3-(4-tert-butylphenyl)-2-methylprop-2-enylidene] malononitrile (DCTB) as matrix. UV-vis spectra were recorded using a Lambda 35 UV-vis spectrometer. FT-IR spectra were obtained on a Nicolet 6700 FT-IR spectrometer. The PL measurement was performed on a QM40 spectrometer. The recycling preparative liquid chromatography is LC-9104. The flow rate was 12 ml/min. Ultrasound treatment was performed on a 20 kHz KC-D40A ultrasonic cleaner at 12–18°C. TEM was performed on a HITACHI HT 7700 electron microscope operating at 95kV accelerating voltage. XRD analysis was performed on a SMARTLAB(3) diffractometer. Cryo-TEM image was obtained at a FEI Talos F200C TEM.

### Synthetic Procedures

**Synthesis of EP3HT.** 2,5-dibromo-3-hexylthiophene (4.034 g, 12 mmol) and THF (48 ml) were added to a 200 ml Schlenk flask. Then isopropylmagnesium chloride (6 ml, 12 mmol) was added to the flask at 0°C and the mixture was stirred for 2 h. After the reaction, the solution was transferred to another 500 ml flask containing 433.6 mg Ni(dppp)Cl_2_ and 96 ml THF. The mixture was then stirred overnight at room temperature. 6.72 ml ethynylmagnesium bromide (0.5 M solution in THF, 3.36 mmol) was added to the flask and the solution was stirred for another 6 h. The reaction was quenched by adding methanol. Filtering could be used to get crude product and the product was purified by Soxhlet extractions with methanol, hexane and chloroform. The hexane extraction was purified by column chromatography (SiO_2_, eluent: chloroform) and circulating preparative liquid chromatography (eluent: Tol) to get the final product. ^1^H NMR (400 MHz, CDCl_3_), δ (ppm): 0.91 (t, 42H, -CH_3_), 1.26–1.43 (m, 84H, -CH_2_-), 1.61 (m, 28H, Ar-CH_2_-CH_2_-), 2.74–2.56 (m, 28H, Ar-CH_2_-), 3.53 (s, 1H, -C≡CH), 6.89–7.06 (s, 14H, Ar-H). GPC: M_n_ = 2,795, PDI = 1.15. MALDI-MS: M_n_ = 1832, PDI = 1.01.

**General procedure for the synthesis of block copolymers.** All block copolymers were prepared by the similar procedure. EP3HT (45 mg, 0.024 mmol), mPEG-N_3_ (413.4 mg, 0.02 mmol) and CuBr (58 mg, 0.4 mmol) were placed in a 50 ml Schlenk flask. 15 ml THF was added to the flask and the mixture was purged with argon for 20 min. After adding 85.2 μl PMDETA (0.4 mmol) to the flask and the solution was purged with argon for another 10 min. The reaction was kept for 2 days at 45°C. After the reaction, the solution was purified by neutral Al_2_O_3_ column twice (eluent: Tol/methanol = 25/1, v/v).

**P3HT-*b*-PEG**: ^1^H NMR (400 MHz, CDCl_3_), δ (ppm): **Triazole ring**: 7.82 (s, 1H, -N_3_HC_2_-). **P3HT block**: 6.98 (s, 10.3H, Ar-H), 2.79–2.57 (m, 21H, Ar-CH_2_-), 1.68 (m, 19.8H, Ar-CH_2_-CH_2_-), 1.25–1.41 (m, 74.6H, -CH_2_-), 0.89 (t, 30.5H, -CH_3_). **PEG block**: 4.5 (t, 2H, -CH_2_-N_3_HC_2_-), 3.79–3.46 (m, 1629H, -OCH_2_CH_2_-), 3.37 (s, 4H, -OCH_3_). GPC: M_n_ = 9761, PDI = 1.77.

**P3HT-*b*-PEG-*b*-P3HT:** ^1^H NMR (400 MHz, CDCl_3_), δ (ppm): **Triazole ring**: 7.82 (s, 2H, -N_3_HC_2_-). **P3HT block**: 6.98 (s, 20.7H, Ar-H), 2.79–2.57 (m, 41.6H, Ar-CH_2_-), 1.68 (m, 42H, Ar-CH_2_-CH_2_-), 1.25–1.41 (m, 120H, -CH_2_-), 0.89 (t, 60H, -CH_3_). **PEG block**: 4.5 (t, 4H, -CH_2_-N_3_HC_2_-), 3.79–3.46 (m, 1714H, -OCH_2_CH_2_-). GPC: M_n_ = 15,582, PDI = 1.56.

**4P3HT-*b*-PEG:** ^1^H NMR (400 MHz, CDCl_3_), δ (ppm): **Triazole ring**: 7.82 (s, 4H, -N_3_HC_2_-). **P3HT block**: 6.98 (s, 40.3H, Ar-H), 2.79–2.57 (m, 78H, Ar-CH_2_-), 1.68 (m, 80H, Ar-CH_2_-CH_2_-), 1.25–1.41 (m, 250H, -CH_2_-), 0.89 (t, 130H, -CH_3_). **PEG block**: 4.5 (t, 9H, -CH_2_-N_3_HC_2_-), 3.79–3.46 (m, 1844H, -OCH_2_CH_2_-). GPC: M_n_ = 15,585, PDI = 1.61. The GPC results were shown in Supplementary Table S1.

### Preparation of Nanocrystal Suspension

The blend solution of block copolymer and C8PDI was heated at 120°C to make the solute dissolve completely. After cooled to room temperature, the solution was exposed to ultrasound for 2 h. The solution was shaken by hand for 0.5 h, and then sonicated for another 1.5 h without shaking. The water temperature of ultrasonic cleaner was maintained at 12–18°C by cooling circulation pump. The nanocrystal suspension of pure block copolymer or C8PDI was prepared by the same method.

### Preparation of TEM Samples

Drop cast 2 μl of suspension on copper grid coated with carbon film and the excess suspension was removed by filter paper. After drying in air, the samples were checked by TEM.

### Preparation of XRD Samples

XRD samples were prepared by drop casting 50–150 μl solutions or colloidal solutions respectively on freshly washed silicon wafers.

## Conclusion

A series of P3HT and PEG block copolymers containing linear diblock, triblock and dendritic star block copolymers were synthesized and found to be able to regulate the crystallization of C8PDI. The regulation effect increases as the P3HT block increases. On the other hand, stable crystalline P3HT-*b*-PEG/C8PDI micelles in water were successfully prepared, which provides a new way to make pollution-free aqueous organic semiconductor inks for printing electronic devices.

## Data Availability

The original contributions presented in the study are included in the article/Supplementary Material, further inquiries can be directed to the corresponding author.
