# Supplementary material for: Crystallization Control of N,N′-Dioctyl Perylene Diimide by Amphiphilic Block Copolymers Containing poly(3-Hexylthiophene) and Polyethylene Glycol
Source: Front Chem. 2021 Jun 10;9:699387. doi: 10.3389/fchem.2021.699387 (PMC8222538; doi:10.3389/fchem.2021.699387)
Supplement: Supplementary file 1 [file DataSheet1.docx]

Supplementary Material

**(A)**


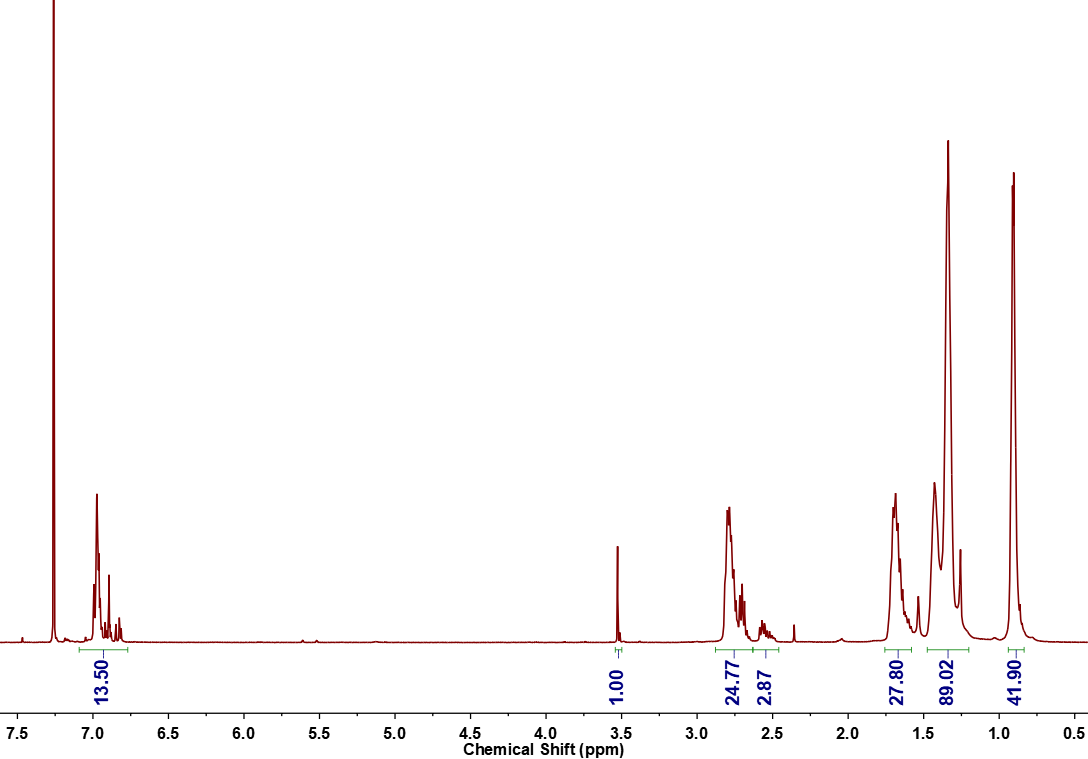


**CDCl_3_**

**7.26**

**a**

**6.92**

**b**

**3.53**

**c**

**2.72**

**d**

**2.53**

**f**

**1.34**

**g**

**0.88**

**e**

**1.64**

**(B)**


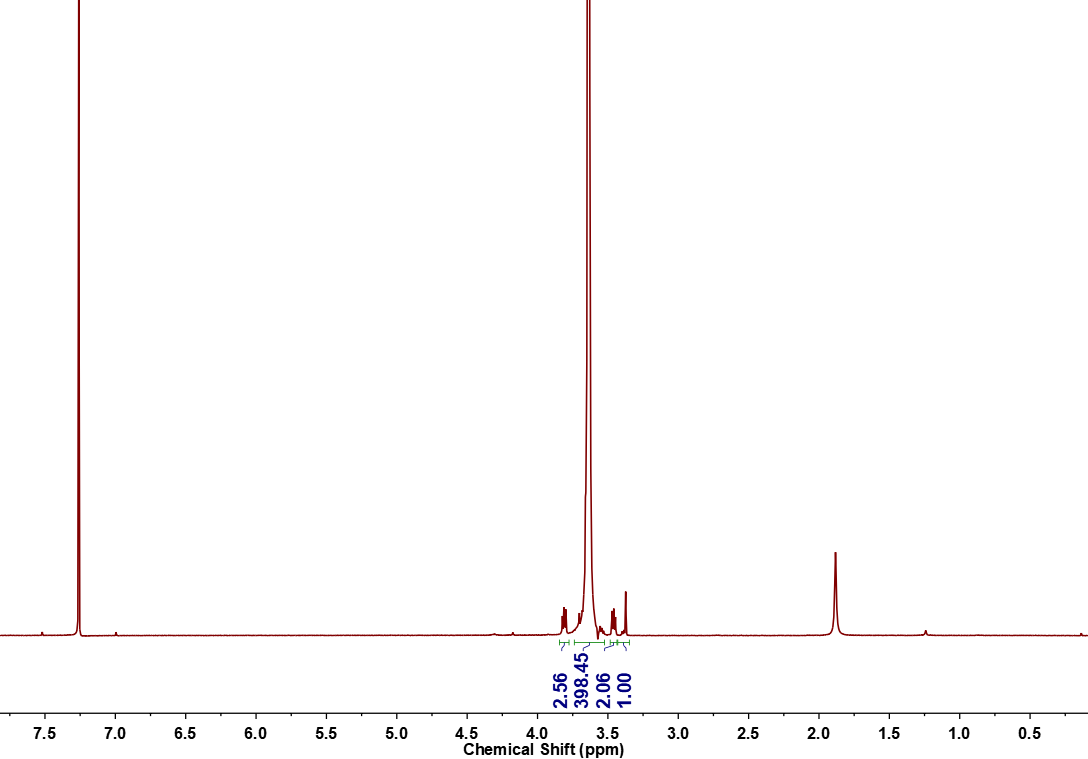


**H_2_O**

**1.88**

**CDCl_3_**

**7.26**

**a**

**3.37**

**b**

**3.64**


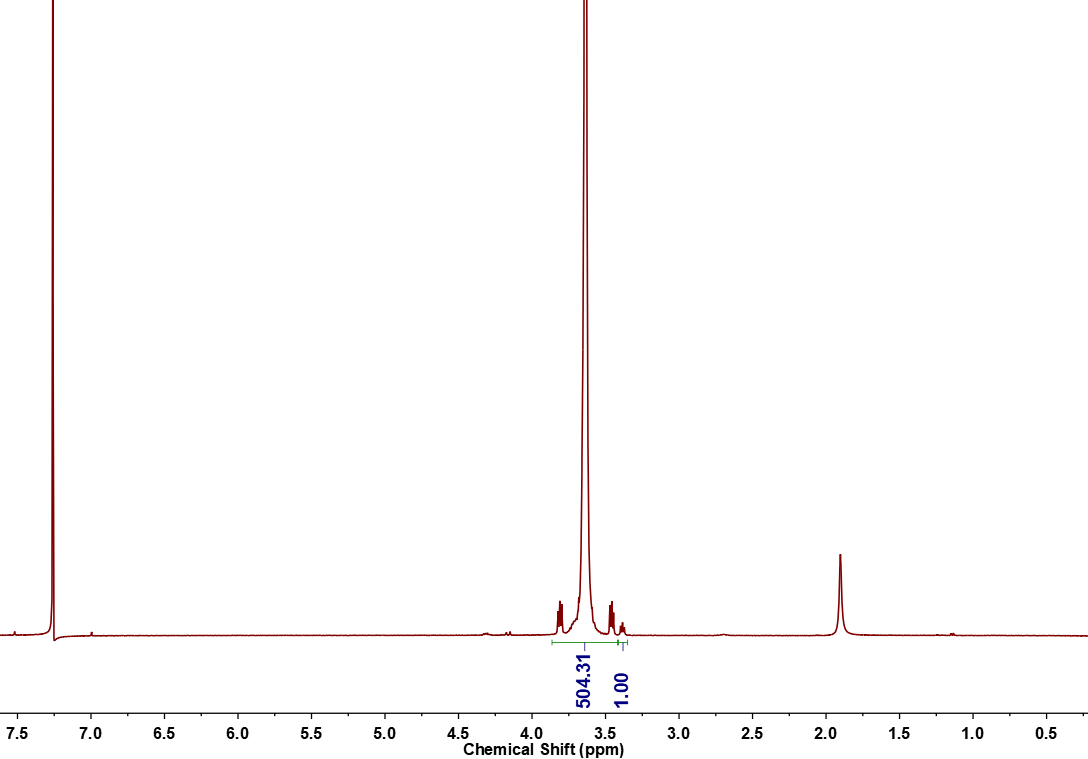


**(C)**

**a**

**3.38**

**CDCl_3_**

**7.26**

**H_2_O**

**1.90**

**b**

**3.64**


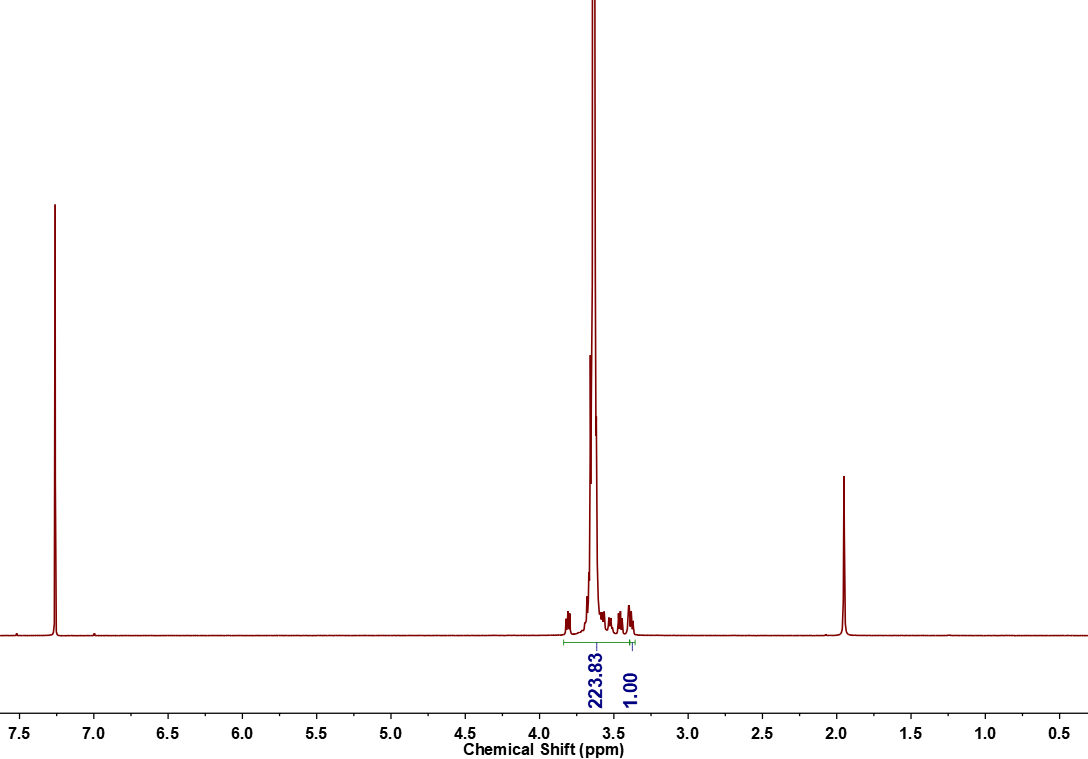


**(D)**

**H_2_O**

**1.95**

**CDCl_3_**

**7.26**

**b**

**3.62**

**a**

**3.38**


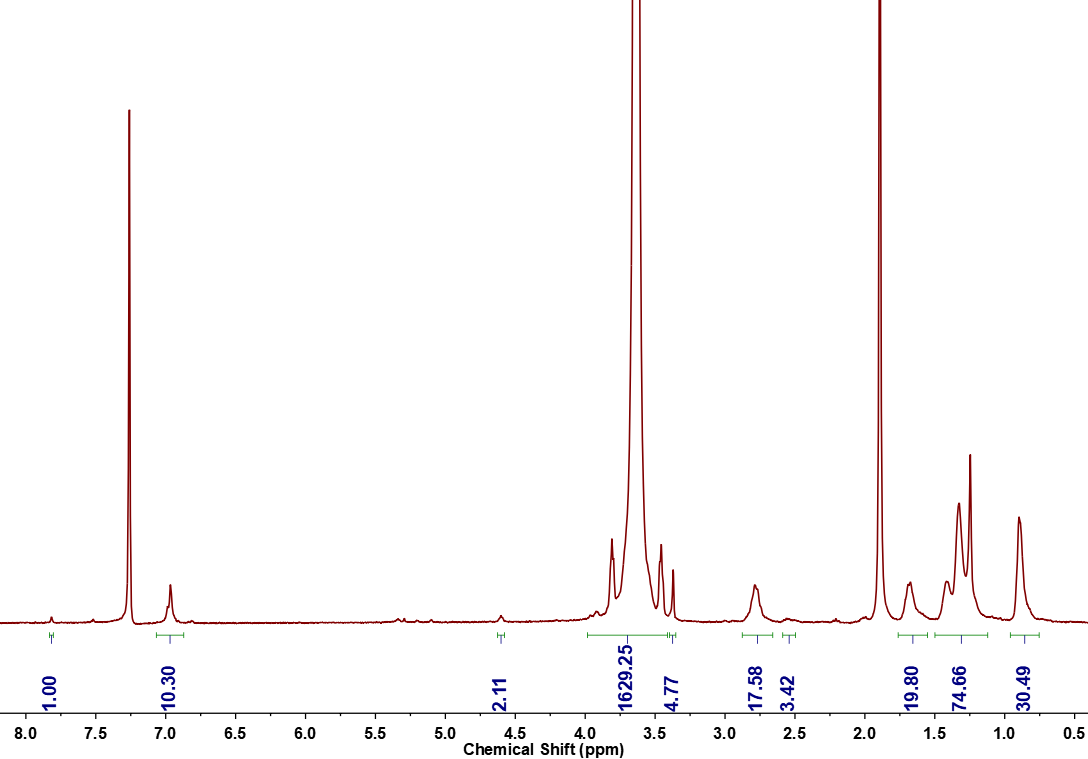


**H_2_O**

**1.89**

**h**

**1.68**

**e**

**3.37**

**f**

**2.78**

**i**

**1.29**

**j**

**0.89**

**(E)**

**d**

**3.63**

**g**

**2.55**

**c**

**4.60**

**b**

**6.98**

**a**

**7.82**

**CDCl_3_**

**7.26**


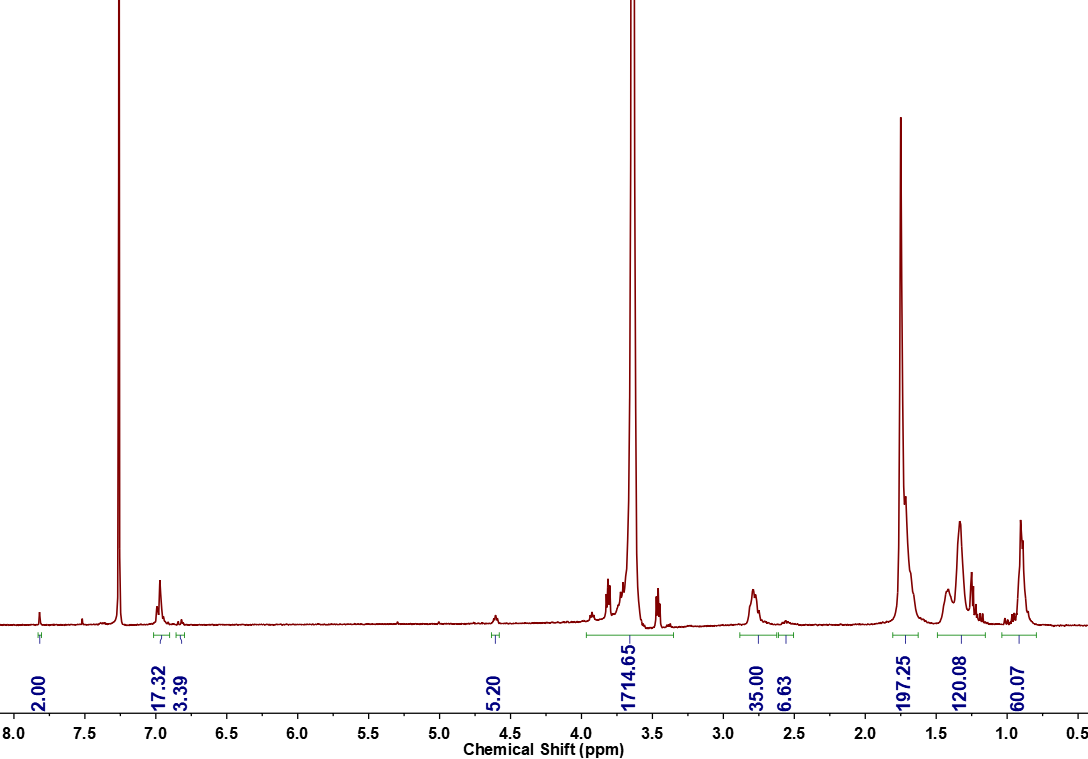


**(F)**

**e**

**2.78**

**c**

**4.61**

**g**

**1.73**

**i**

**0.92**

**h**

**1.30**

**f**

**2.55**

**d**

**3.64**

**b**

**6.98**

**a**

**7.82**

**CDCl_3_**

**7.26**


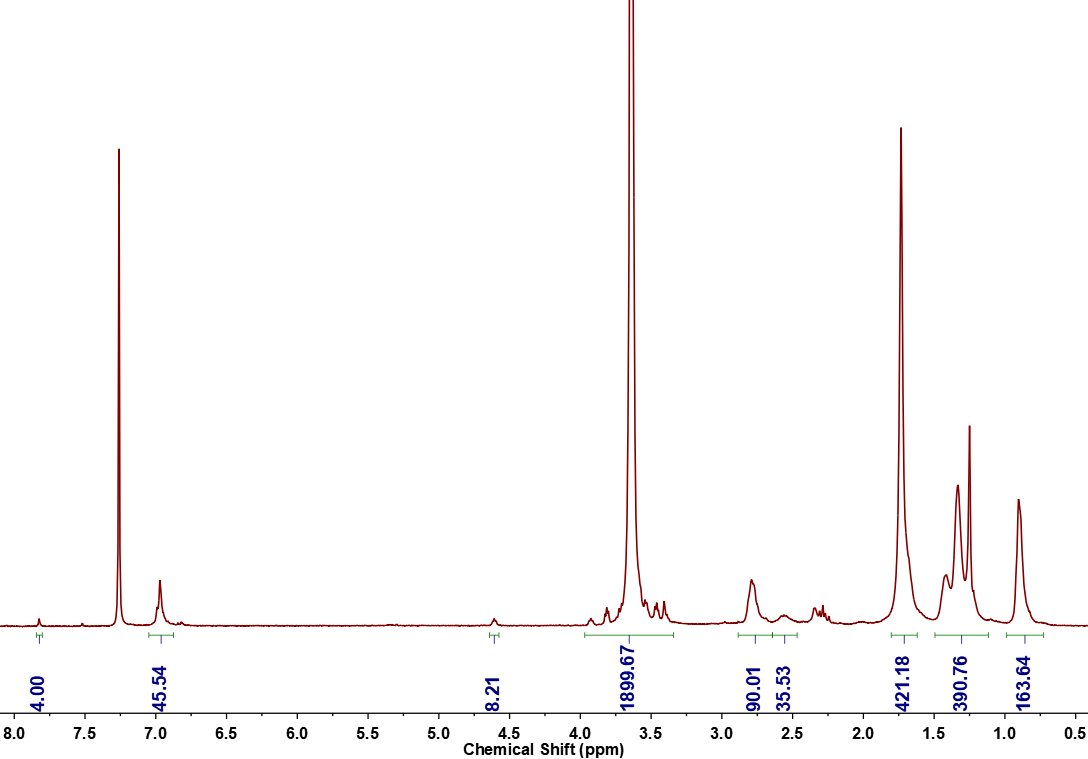


**CDCl_3_**

**7.26**

**a**

**7.82**

**b**

**6.98**

**c**

**4.61**

**d**

**3.64**

**e**

**2.78**

**f**

**2.55**

**g**

**1.71**

**h**

**1.29**

**i**

**0.88**

**(G)**

**Figure S1.** ^1^H NMR spectra of EP3HT (A), mPEG-N_3_ (B), N_3_-PEG-N_3_ (C), 4PEG-N_3_ (D), P3HT-*b*-PEG (E), P3HT-*b*-PEG-*b*-P3HT (F) and 4P3HT-*b*-PEG (G).

**Figure S2.** GPC curves of EP3HT, mPEG-N_3_ and P3HT-*b*-PEG in THF.

**Figure S3.** GPC curves of EP3HT, N_3_-PEG-N_3_ and P3HT-*b*-PEG-*b*-P3HT in THF.

**Figure S4.** GPC curves of EP3HT, 4PEG-N_3_ and 4P3HT-*b*-PEG in THF.


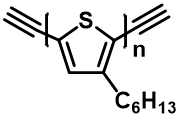


95% Intensity

5% Intensity

**Figure S5.** MALDI-TOF-MS spectrum of EP3HT.

**Figure S6.** FT-IR spectra of mPEG-N_3_, N_3_-PEG-N_3_ and 4PEG-N_3_.


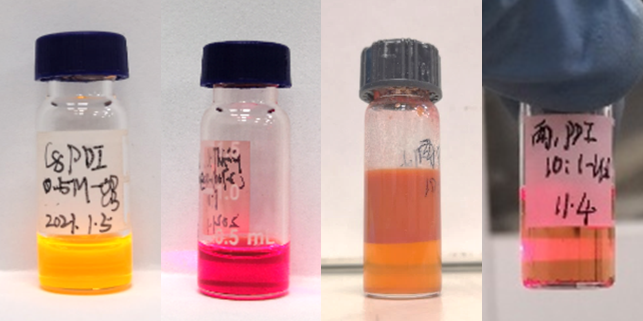


**(A)**

**(B)**

**(C)**

**Figure S7.** Pictures of Tyndall effect phenomenon of *o*-DCB solution of P3HT-*b*-PEG and C8PDI (1:0.5, A), layering phenomenon after adding water to extract the sonicated Tol solution of P3HT-*b*-PEG and C8PDI (10:1, B), and Tyndall effect phenomenon of the water phase extracted from the sonicated Tol solution of P3HT-*b*-PEG and C8PDI (10:1, C).


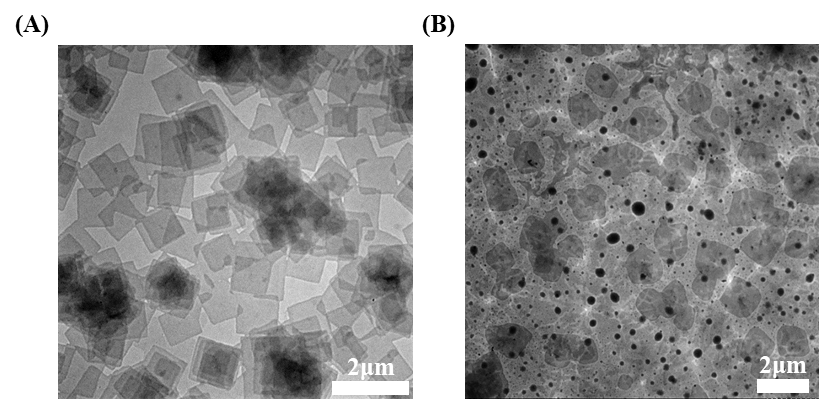


**Figure S8.** TEM images of P3HT-*b*-PEG drop-cast from Tol solution (10 mg/mL) after ultrasound (A) and mPEG-N_3_ drop-cast from Tol solution (10 mg/mL) after ultrasound (B).

**Table S1.** M_n_, M_w_ and PDI of EP3HT, PEG-N_3_ and block copolymers.

| **Samples** | **M_n_ (g/mol)** | **M_w_ (g/mol)** | **PDI** |
| --- | --- | --- | --- |
| EP3HT | 2800 | 3200 | 1.15 |
| mPEG_450_-N_3_ | 15800 | 21600 | 1.37 |
| N_3_-PEG-N_3_ | 11300 | 18600 | 1.65 |
| 4PEG-N_3_ | 15200 | 21500 | 1.42 |
| P3HT-*b*-PEG | 9700 | 17300 | 1.77 |
| P3HT-*b*-PEG-*b*-P3HT | 15600 | 24400 | 1.56 |
| 4P3HT-*b*-PEG | 15600 | 25200 | 1.61 |
